# Construction and osteogenic effects of 3D-printed porous titanium alloy loaded with VEGF/BMP-2 shell-core microspheres in a sustained-release system

**DOI:** 10.3389/fbioe.2022.1028278

**Published:** 2022-10-21

**Authors:** Zheng Liu, Zhenchao Xu, Xiyang Wang, Yilu Zhang, Yunqi Wu, Dingyu Jiang, Runze Jia

**Affiliations:** ^1^ Department of Orthopedics, Hunan Children’s Hospital, Changsha, Hunan, China; ^2^ Department of Spine Surgery and Orthopaedics, Xiangya Hospital, Central South University, Changsha, Hunan, China; ^3^ Hunan Engineering Laboratory of Advanced Artificial Osteo-Materials, Xiangya Hospital, Central South University, Changsha, Hunan, China

**Keywords:** shell-core microspheres, 3D-printed porous titanium alloy, composite scaffold, osteogenic differentiation, osseointegration

## Abstract

The repair and reconstruction of bone defects remain a challenge in orthopedics. The present study offers a solution to this problem by developing a vascular endothelial growth factor (VEGF)/bone morphogenetic protein 2 (BMP-2) shell-core microspheres loaded on 3D-printed porous titanium alloy *via* gelatin coating to prepare a titanium-alloy microsphere scaffold release system. The composite scaffold was characterized *via* scanning electron microscope (SEM) and energy disperse spectroscopy (EDS), and the effect of the composite scaffold on the adhesion, proliferation, and differentiation of osteoblasts were determined *in vitro*. Furthermore, a rabbit femoral defect model was established to verify the effect of the composite scaffold on osteogenesis and bone formation *in vivo*. The results demonstrated that the composite scaffold could release VEGF and BMP-2 sequentially. Meanwhile, the composite scaffold significantly promoted osteoblast adhesion, proliferation, and differentiation (*p* < 0.05) compared to pure titanium alloy scaffolds *in vitro*. Furthermore, the composite scaffold can exhibit significant osteogenic differentiation (*p* < 0.05) than gelatin-coated titanium alloy scaffolds. The *in vivo* X-rays demonstrated that the implanted scaffolds were in a good position, without inflammation and infection. Micro-CT and quantitative results of new bone growth illustrated that the amount of new bone in the composite scaffold is significantly higher than that of the gelatin-coated and pure titanium alloy scaffolds (*p* < 0.05). Similarly, the fluorescence labeling and V-G staining of hard tissue sections indicated that the bone integration capacity of the composite scaffold was significantly higher than the other two groups (*p* < 0.05). This research suggests that VEGF/BMP-2 shell-core microspheres loaded on 3D-printed titanium alloy porous scaffold through gelatin hydrogel coating achieved the sequential release of VEGF and BMP-2. Most importantly, the *in vitro* and *in vivo* study findings have proven that the system could effectively promote osteogenic differentiation and osseointegration.

## 1 Introduction

Titanium (Ti) and the associated alloys are currently preferred for orthopedic implants due to their excellent biocompatibility and mechanical properties (Xu et al., 2022). Selective laser melting (SLM) is a three-dimensional (3D) printing technology; The metal powder is completely melted, cooled, solidified, and metallurgically welded with the base metal under a high-energy laser before the accumulated layer by layer to form a 3D solid (Cassetta et al., 2015). For instance, the 3D-printed titanium alloy scaffold has a 3D porous structure, providing necessary new bone growth and blood vessel formation. Meanwhile, the 3D-printed titanium alloy scaffold design is patient-specific to improve osseointegration, where the implant has a highly controllable internal structure and customized surface features (Zhang et al., 2018). The chemical stability and structure of the oxide layer on the titanium surface are responsible for the excellent surface properties. Despite all the potential benefits of the oxide layer, the osseointegration ability or biological activity of titanium has not been thoroughly explored to achieve chemical bonding between the implant and bone tissue. Ti and bones are generally separated by a 10-nm-thick non-mineral layer (Vanderleyden et al., 2014). The modification of the Ti surface may promote cell adhesion, avoid excessive fretting of implants, and reduce fibrous tissue formation. Consequently, the integration of the implant-bone interface can be facilitated, mechanical processing stability can be improved, and the risk of implant loosening can be reduced, accelerating bone repair.

BMP-2 and VEGF are critical in osteogenesis and differentiation, besides complementing each other in biological function and accelerating bone formation (Jung et al., 2017; Divband et al., 2021). The former promotes osteogenesis and bone formation, whereas the latter stimulates angiogenesis in bone formation. Thus, combining the two growth factors can enhance bone repair (Geng et al., 2021). Given the complex and highly coordinated time and course of bone defect regeneration, a time-coupled scaffold mimics osteogenesis and angiogenesis and could also facilitate bone defect repair (Eğri and Eczacıoğlu, 2017; Sharmin et al., 2017; Dou et al., 2019). Several encapsulation technologies have been developed to achieve the time release of different growth factors (Amini et al., 2013; de Matos et al., 2013; Yang et al., 2016). The core-shell microspheres are one of the most attractive options for sequential delivery of growth factors due to the multi-purpose drug delivery method and easy integration into many scaffold matrices.

The coating of biological materials or growth factors has been demonstrated to strengthen bone regeneration and shorten osseointegration time, resulting in improved initial implant stability (Spriano et al., 2018). Gelatin is a naturally available and inexpensive material with excellent biocompatibility, biodegradability, and low immunogenicity. Furthermore, gelatins possess the properties and characteristics of hydrogels, hence suitable for surface coating in gel form or combined with other materials to produce a composite scaffold (Liu et al., 2019). Ultimately, gelatin is ideal for developing tissue engineering and drug delivery biomaterials.

The repair and reconstruction of bone defects remain a challenge in orthopedics. In order to avoid the potential shortcomings of autologous bone and allogeneic bone, bone repair scaffolds are considered as the trend of clinical application. The key strategy to design composite bone repair scaffolds is to simulate the natural bone healing process. In this study, the VEGF/BMP-2 core-shell sustained-release microspheres were immobilized on 3D-printed porous titanium alloy scaffolds *via* gelatin gel coating to prepare a 3D-printed porous titanium alloy scaffold, loaded with VEGF/BMP-2 core-shell microspheres for a sustained-release system. In addition, the characterization and biocompatibility of this component were evaluated, and the osseointegration effects were investigated *in vitro* and *in vivo*.

## 2 Materials and methods

### 2.1 Preparation of 3D-printed porous titanium alloy by selective laser melting technology

First, the 3D data models of porous titanium alloy scaffolds with hollow hexagonal prism were designed using the Solid Works CAD software (Dassault Systèmes, France). Second, the designed scaffolds were established from Ti6Al4V powder (TC4) (Research Institute of Powder Metallurgy, Central South University, China) using metal laser melting equipment (Hunan Farsoon High-Technology Co., LTD., China). The un-melted metal powder in the scaffold was then removed with an air gun, followed by continuous ultrasonic cleaning for 10 min using acetone (Sinopharm, China), anhydrous ethanol (Sinopharm, China), and deionized water. Subsequently, porous titanium alloy scaffolds were placed in a 5 mol/L NaOH solution at 60°C for 24 h and repeatedly cleaned with deionized water before drying. The porous scaffolds were then placed in 0.5 mmol/L HCl solution at 70°C for 24 h and cleaned with deionized water before drying. Finally, the scaffolds were placed in a sintering furnace (FCT Systems, Germany) at 600°C for 5 °C/min, followed by atmospheric annealing for 1 h. Porous titanium alloy scaffolds are available in two specifications, one with a diameter of 10 mm and a height of 5mm, and the other with a diameter of 5 mm and a height of 10 mm. The porosity is 60% and the pore size is 500 μm.

### 2.2 Preparation of VEGF/BMP-2 shell-core microspheres by coaxial electrostatic spray technology

A volume of 10 μg/ml VEGF (Sigma-Aldrich, United States) in deionized water, 100 μg/ml BMP-2 (Sigma-Aldrich, United States) in deionized water, polylactic-co-glycolic acid (PLGA, Av. Mol. Wt, 40,000 Da) (Sigma-Aldrich, United States) in ethyl acetate (5%, w/v), and Poly (L-lactic acid) (PLLA, Av. Mol. Wt, 90,000 Da) (Sigma-Aldrich, United States) in dichloromethane (5%, w/v) were prepared. The growth factor solutions served as the aqueous phases and the polymer solutions as the organic phases. The aqueous and organic solutions were mixed in a 1:10 ratio and placed in an ultrasonic shaker until a homogeneous emulsion was formed. The polymer concentration was set at 5%, and the flow rates of the solutions in the inner and outer tubes of the coaxial needle were set at 1.5 ml/h, with a voltage of 10.0 kV.

The injection pumps for the PLGA and PLLA solutions were connected to the outer and inner needle tubes of the coaxial needle, respectively. The electrospinning process was then initiated, and the voltage was adjusted progressively. The microspheres were collected about 15 cm below the coaxial needle tip in a glass vessel filled with an appropriate amount of absolute ethanol. The collected anhydrous alcohol microsphere suspension was transferred to a 50 ml centrifuge tube, centrifuged for 3 min at 175 × *g*, followed by washing with double steamed water thrice after removing the ethanol from the upper layer. Finally, the solution was incubated at 80°C for 30 min, freeze-dried for 72 h, and stored in the refrigerator at 4°C away from light until use. For observation of the surface morphology of the core-shell microspheres by scanning electron microscope (SEM) (Czech FEI Company), freeze-dried microspheres were sprayed with gold for 60 s and then observed.

### 2.3 Preparation of a 3D-printed porous titanium alloy scaffold-loaded VEGF/BMP-2 shell core microsphere sustained-release system

**FIGURE 1:**
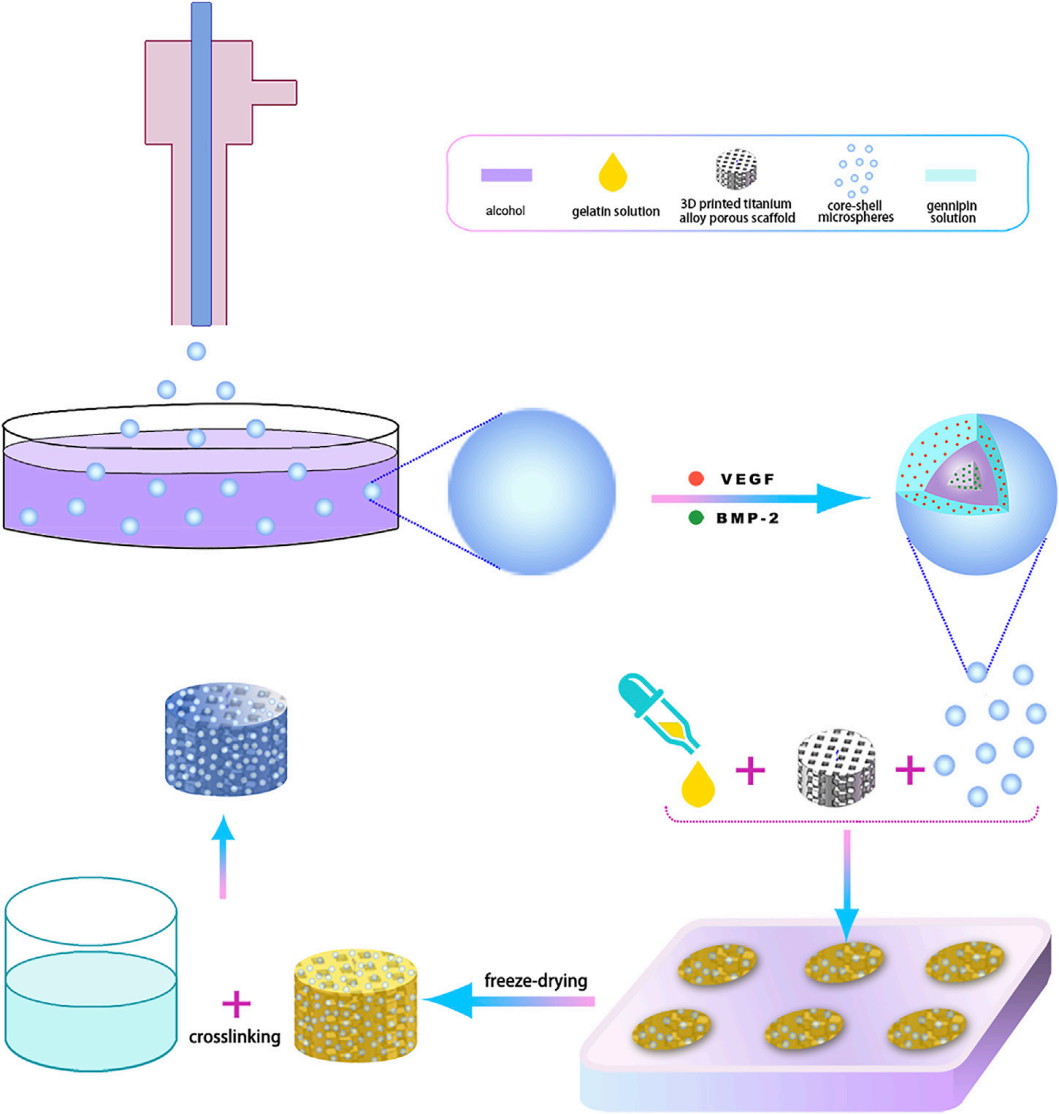
The 3D-printing process diagram of titanium alloy porous scaffolds loaded with two-factor core-shell microspheres sustained-release system.

A total of 600 mg gelatin (Dalian Meilun Biotechnology Co., LTD., China) was added to 10 ml of deionized water and stirred at room temperature until transparent. The pre-prepared 3D printed hollow, hexagonal, titanium alloy porous scaffold was slotted into a custom-made polytetrafluoroethylene (PTFE) holder. Subsequently, 20 mg of VEGF/BMP-2 shell-core microspheres and 500 ul of gelatin solution were injected into the PTFE holder fitted with scaffold and mixed thoroughly, pre-frozen at −80°C, followed by freeze-drying for 72 h and cross-linked with 1% genipin (Dalian Meilun Biotechnology Co., LTD., China) at –37°C for 12 h. The mixture was later washed with absolute ethanol thrice for 1 h each session. Finally, the 3D-printed porous titanium alloy scaffold-loaded VEGF/BMP-2 shell-core microsphere sustained-release system was obtained after drying. The preparation process is illustrated in Figure 1.

### 2.4 Characterization of 3D printed porous titanium alloy scaffold loaded VEGF/BMP-2 shell core microsphere sustained release system

The prepared composite scaffolds were subjected to gold-spray treatment. The surface morphology of the scaffolds was then observed by SEM. Similarly, the flat structural area of the titanium alloy porous scaffolds in each group was subjected to SEM. Meanwhile, EDS (Bruke Germany Company) was used to analyze the elements on the scaffold surface.

### 2.5 *In vitro* experiments of 3D-printed porous titanium alloy scaffold-loaded VEGF/BMP-2 shell-core microspheres sustained-release system

The composite scaffolds were placed in the dialysis bag, followed by a centrifuge tube containing 10.0 ml PBS. The centrifuge tube was then placed in a constant temperature incubator (37°C, 120 rpm). The PBS was aspirated from the centrifuge tube periodically at the indicated time point (on days 1, 2, 4, 6, 8, 11, 14, 18, 22, and 27) and immediately replaced with 5 ml of fresh PBS. The recovered PBS was stored at –20°C. The concentrations of BMP-2 and VEGF recovered at each time point were determined using ELISA kits (Wuhan Huamei Biological Engineering Co., LTD., China). The release amount of BMP-2 and VEGF were calculated, and the release curves were plotted.

### 2.6 *In vitro* biocompatibility study of 3D-printed titanium alloy porous scaffold-loaded VEGF/BMP-2 shell-core microspheres sustained-release system

The *in vitro* study was conducted using three groups, including dual-factor loaded core-shell microspheres-gelatin coating-3D printing titanium alloy porous scaffold (Group A), gelatin coating-3D printing titanium alloy porous scaffold (Group B), and 3D printed titanium alloy porous scaffolds (Group C/control group). The 3D printed porous titanium alloy selected are 10 mm in diameter and 5 mm in height *in vitro* experiments.

### 2.7 Cell suspension preparation

The MC3T3-E1 osteogenic precursor cell suspension (Cell Resource Center, Shanghai Institutes of Biological Sciences, Chinese Academy of Sciences, China) was cultured in the α-MEM medium containing 10% fetal bovine serum (Gibco, United States) and 1% double antibodies (streptomycin and penicillin at 100 U/mL; Hyclone, United States). The culture plate was placed in an incubator at 37°C with 5% carbon dioxide (CO_2_), and the culture medium was changed every 2 days. Cells were digested by passage and counted with a cell counter. The culture medium was later added to prepare the required concentration of cell suspension.

### 2.8 Observation of cell morphology on the scaffold surface by scanning electron microscopy

The scaffolds of each groups were disinfected and placed in 48-well plates in triplicates. First, the cell suspension with a concentration of 2×10^6^/ml was extracted, and 100 μl cell suspension per well was inoculated on the material surface. Subsequently, 700 µl of cell suspension was added to the medium to cover the material surface. The inoculated 48-well culture plate was then placed in an incubator at 37°C and 5% CO_2_, and the culture medium was changed every 2 days.

After 48 h of co-culture, the medium was discarded. Three times washings later, the PBS was aspirated, discarded, and fixed in the pre-cooled 2.5% glutaraldehyde for 4 h at 4°C. The glutaraldehyde was then aspirated and discarded. Subsequently, dehydration was performed twice in a series of ethanol (50%, 60%, 70%, 80%, 90%, and 100%). After the surface was sprayed with gold, the surface morphology was observed by SEM.

### 2.9 Observation of cell morphology on the scaffold surface by confocal laser microscopy

The cell suspension with a concentration of 2×10^6^/ml was co-cultured with scaffolds of each group. After cells and scaffolds were co-cultured for 24 h, the culture plate was removed, and the medium was aspirated and discarded. The scaffolds were then washed with PBS thrice and gently placed into a new 24-well plate with microscopic tweezers. A volume of 1 ml of 4% paraformaldehyde solution was added to each well for 5 min to fix the scaffolds. The fixed solution was later aspirated and discarded, and the scaffolds were rinsed with PBS. Subsequently, 0.3% Triton-X-100 solution was added to break the membrane at a constant temperature for 15 min. The membrane-breaking solution was aspirated and discarded, followed by blocking with 5% BSA for 1 h. After discarding the blocking solution, the medium was rinsed with PBS, which was later discarded.

Drops of phalloidin staining solution (50 μg/ml) were gently added to the scaffold surface. The scaffold surface should be completely covered with the staining solution, and the staining was performed at room temperature for 40 min in the dark. The staining solution was then removed, and the cells were washed with PBS thrice for 10 min each on the shaking table. After washing, the liquid was aspirated and discarded. A 50 μg/ml 4′,6-diamidino-2-phenylindole (DAPI) solution was dripped onto the scaffold surface to redye the nucleus for 5 min. Subsequently, PBS solution was added, shaken, and washed thrice for 10 min each session, and the whole redyeing process was conducted away from light. Finally, the culture dish was placed under confocal laser microscopy (CLSM) (Zeiss, Germany) to observe the morphology of the cell cytoskeleton.

### 2.10 Effect of scaffolds on cell proliferation

The cell suspension with a concentration of 2×10^6^/ml was co-cultured with scaffolds of each group. On the 1st, 3rd, 5th, and 7th day of the culture, the CCK8 reagent (DOJINDO, Japan) and the culture solution were mixed and diluted in a 1: 10 ratio, and the culture solution of each well was discarded. The diluted CCK-8 reagent (800ul) was added to each well and further cultured in the incubator for 4 h. The supernatant of each well was then carefully collected and transferred to a 96-well plate. Finally, the absorbance (OD value) was measured with a microplate reader at a wavelength of 450 nm and recorded.

### 2.11 Quantitative detection of ALP activity

The cell suspension with a concentration of 5×10^6^/ml was co-cultured with scaffolds of each group. After 1 day of co-culture, the medium was changed to osteogenic induction medium (α-MEM + β-sodium glycerophosphate + ascorbic acid + dexamethasone) and placed in a cell culture incubator for co-culture. The medium was changed every 3 days. On the 12th day of co-cultivation, the medium was aspirated, and the cells were gently rinsed thrice with PBS. Typically, 1% Triton X-100 in PBS buffer (200 µl) was added to each well to lyse the cells and the supernatant was collected *via* centrifugation. Finally, ALP activity was quantitatively detected with an ALP detection kit (Beyotime Biotechnology, China).

### 2.12 Quantitative detection of alizarin red staining

The cell suspension with a concentration of 5×10^6^/ml was co-cultured with scaffolds of each group. The medium was aspirated on the 21st day of co-cultivation in the osteogenic medium. The cells were washed thrice with PBS and fixed with 4% paraformaldehyde for 30 min. The fixation solution was aspirated and discarded; then, the cells were rinsed thrice with PBS. The alizarin red staining solution (Cyagen Biosciences, United States) was added to the cultures at 37°C for 30 min. Subsequently, the alizarin red staining solution was aspirated and discarded, while the supernatant was washed with deionized water until colorless. Chlorohexadecylpyridine (10%, solarbio Biotechnology Co., Ltd., China) was added to each well. After the alizarin red was dissolved entirely at room temperature, it was added to a 96-well plate, and OD values were measured at a wavelength of 620 nm.

### 2.13 Detection of osteogenesis-related genes by quantitative real-time polymerase chain reaction

The cell suspension with a concentration of 5×10^6^/ml was co-cultured with scaffolds of each group. On the 14th day of co-culture in the osteogenic medium, cells were washed with PBS, digested by trypsin, centrifuged at 12,000 rpm/min at 4°C for 5 min, and the supernatant was removed. The cells were then gently rinsed with PBS and collected for RNA extraction. Total RNA from each group was extracted and reverse transcribed into cDNA and mRNA expression levels of Runt-related transcription factor 2 (RUNX2), osteocalcin (OCN), and osteopontin (OPN) were detected *via* real-time quantitative real-time polymerase chain reaction (PCR). The primer sequences are shown in Table 1.

**TABLE 1:** Primer sequences used for detection of osteogenesis-related gene expression.

Genes	Primers	Primer sequence (5′-3′)	Product length (bp)
Actin	Forward	ACA​TCC​GTA​AAG​ACC​TCT​ATG​CC	223
	Reverse	TAC​TCC​TGC​TTG​CTG​ATC​CAC	
RUNX-2	Forward	CTC​CTC​TGT​CCC​GTC​ACC​T	135
	Reverse	ATC​ACA​ACA​GCC​ACA​AGT​TAG​CG	
OCN	Forward	CTG​ACC​TCA​CAG​ATG​CCA​A	192
	Reverse	CAT​ACT​GGT​CTG​ATA​GCT​CGT	
OPN	Forward	GAG​GAA​ACC​AGC​CAA​GGT​AA	117
	Reverse	CCA​AAC​AGG​CAA​AAG​CAA​AT	

The qPCR reaction conditions were as follows: denaturation: 95°C for 10 min, 40 cycles at 95°C for 15 s, and 60°C for 30 s. The PCR was performed for 40 cycles, followed by a final extension at 72°C for 10 min; Actin was used as the internal reference gene. The final products were subjected to electrophoresis on 1.5% agarose gel.

### 2.14 Detection of osteogenesis-related proteins by Western blot

The cell suspension with a concentration of 5×10^6^/ml was co-cultured with scaffolds of each group. On day 21 of co-culture in osteogenic medium, cells were washed with PBS, digested with trypsin, centrifuged at 12,000 rpm/min for 5 min at 4°C, and the supernatant was removed. Cells were gently rinsed with PBS and collected for protein extraction. Subsequently, lysis buffer was added to lyse fully, and protein concentration was determined *via* BCA standard curve method. A series of operations were performed, including dispensing, spotting, electrophoresis, cutting glue, membrane cutting, and membrane transferring.

The nitrocellulose membrane section and filter paper were positioned from the negative electrode to the positive electrode and blocked for 1 h. Then, the primary antibodies, RUNX2, OPN, and β-actin (Proteintech Group, Inc., United States), were incubated overnight at 4°C with a dilution ratio of 1:1,000 and incubated with the secondary antibodies at a dilution ratio of 1:10,000 for 2 h at room temperature. The membranes were then washed with Tris Buffered Saline Tween (TBST) thrice for 10 min each. After TBST washing and enhanced chemiluminescence (ECL) development for 1 min, the membrane was exposed under X-ray. Finally, the film was scanned, and the fine absorbance and molecular weight of the band were analyzed.

### 2.15 *In vivo* pro-osteogenic effect of 3D-printed titanium alloy porous scaffold-loaded VEGF/BMP-2 shell-core microspheres sustained-release system

#### 2.15.1 Construction of rabbit femoral condyle defect model

General grade New Zealand male rabbits (Hunan Taiping Biological Technology Co. LTD., China), at 5 months old with an average of 3 kg per animal, were used in this experiment. The 18 male, New Zealand rabbits, were randomly divided into three groups, with six animals in each group as follows: Group A: Dual-factor-loaded shell-core microspheres-gelatin coating 3D-printed titanium alloy porous scaffold; Group B: Gelatin coating 3D-printed titanium alloy porous scaffold; Group C: 3D-printed titanium alloy porous scaffold (control group). After weighing the animals, the New Zealand rabbits were anesthetized with 3% pentobarbital sodium (1 ml/kg) through the marginal ear vein. Following successful anesthesia, most of the hair around the left femoral condyle was shaved.

Anesthetized rabbits were restrained in the right lateral recumbence position on a surgical table. Routine disinfection was conducted, and towels were spread to prepare for surgery. Following a longitudinal incision at the left distal lateral femur, the periosteum was removed to expose the lateral femoral condyle. A mark was made approximately 4 mm from the articular surface, and the hole was re-tapped with an electric drill to create a cylindrical bone defect area (diameter = 5 mm, depth = 10 mm). The surgical site was cooled by washing with saline during drilling. After drilling, the bone defect area was washed with hydrogen peroxide and normal saline, and the scaffolds of each group were implanted into the defect area. The incision was sutured layer by layer, disinfected with iodine, and cefoxitin was injected 3 days after the operation. The 3D printed porous titanium alloy specifications for *in vivo* experiments are 5 mm in diameter and 10 mm in height.

Animals to be sacrificed were injected with calcein solution (solarbio Biotechnology Co., Ltd., China) on day 14 and 4 days before the postoperative harvest. Calcein (400 mg) was dissolved in 50 ml of physiological saline, and 1 g of sodium bicarbonate was added to ensure that the dye was completely dissolved before being subjected to sterilization filtration. At four and 12 weeks post-operation, the animals were sacrificed *via* intravenous injection of an excessive anesthetic. The left femoral condyle was removed, and the surface soft tissue was shaved, fixed with 80% alcohol, and stored at 4°C in the dark.

### 2.16 X-rays and micro-CT examinations

The plain X-ray films of specimens in each group were examined and scanned using micro-CT at a voltage of 70 kV, a current of 141 μA, a scanning range of 360°, and a tomographic thickness of 18 um. The Ctan software was used for 3D reconstruction, and the area within the bottom surface of the scaffold was selected as the region of interest (ROI). New bone tissue in porous scaffold pores was identified by grey scale analysis, and the following parameters were calculated: 1) Trabecular thickness (Tb.Th); 2) Trabecular number (Tb.N); 3) Bone volume fraction (BV/TV) = Bone volume (BV)/Total volume (TV).

### 2.17 Hard tissue biopsy and picric acid fuchsin (Van Gieson, VG) staining

After completion of the micro-CT examination, all samples were immersed in 10% formalin solution for 24 h, 70% alcohol for 2 h, 95% alcohol for 20 h, and 100% alcohol for 2 h, followed by drying under reduced pressure for 6 h. Subsequently, all samples were immersed in a glass tube filled with polymethyl methacrylate monomer, impregnated under a low vacuum for 30 min (no gas bubbling at this time), and placed in a 27°C water bath for curing. The samples were sectioned (200 μm) using a hard tissue microtome and adhered to the resin sheet with quick-drying glue, and the sample slices on the resin sheet were ground to 50 μm with a grinder and then polished. Finally, the fluorescent labeling of bone tissue was observed under CLSM and stained with VG. Images were observed under an optical microscope and collected. The area of new bone tissue in the scaffold was semi-quantitatively analyzed by ImageJ software (NIH, United States), and the calculation method was as follows: the percentage of new bone area fraction in each visual field (%) = new bone area/total visual field area × 100%.

### 2.18 Statistical analysis

All data were analyzed using the statistical package for the Social Sciences (SPSS) 24.0 system (IBM, United States), and the data were expressed as mean ± standard deviation (X ± S). The independent sample *t*-test was used to analyze differences among groups. The *P*- value for each analysis was calculated, and *p* < 0.05 was considered statistically significant.

## 3 Results

### 3.1 Preparation results of VEGF/BMP-2 shell-core microsphere sustained-release system loaded with a 3D printed titanium alloy porous scaffold

**FIGURE 2:**
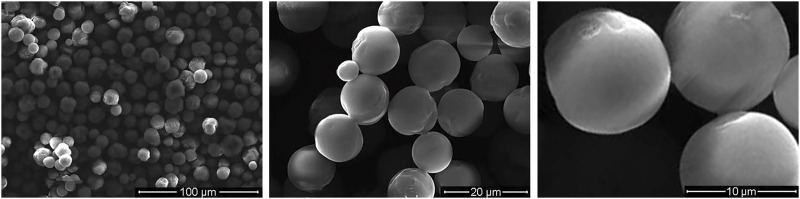
SEM photos of core-shell microspheres.

**FIGURE 3:**
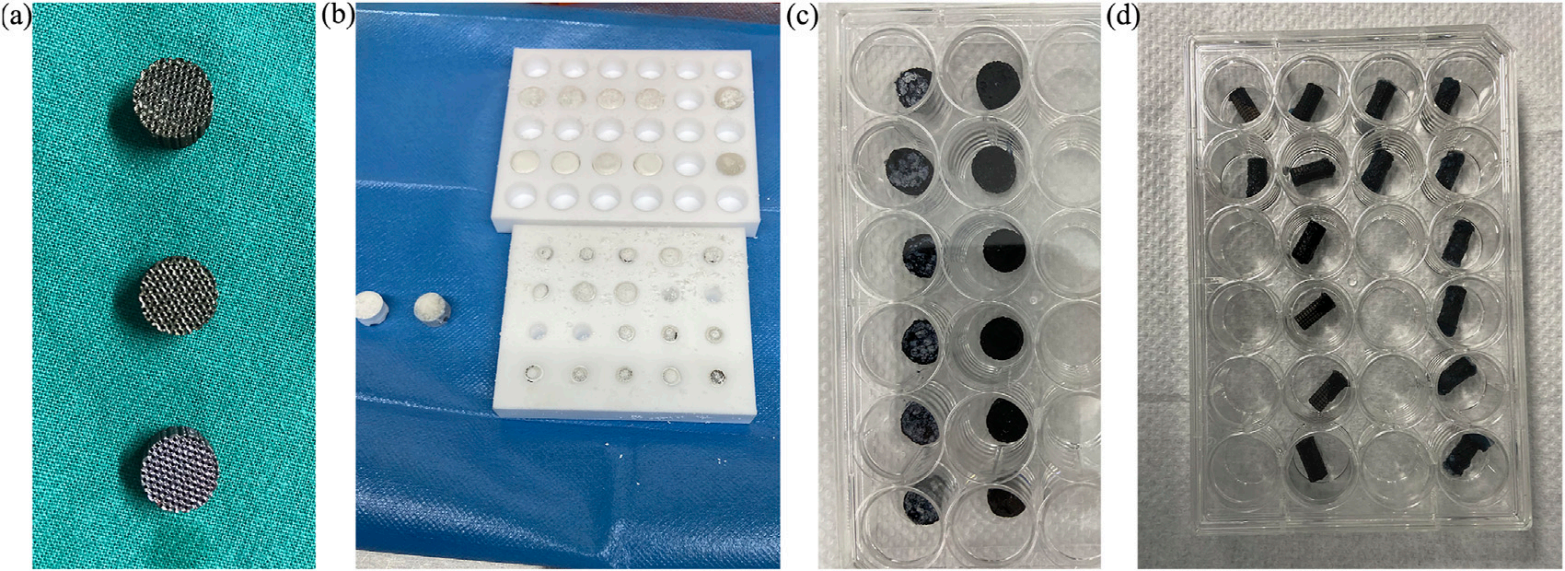
**(A)** 3D-printed titanium alloy porous scaffold; **(B)** freeze-dried scaffold; **(C,D)** composite scaffold after cross-linking.

Figure 2 shows the microspheres had a regular morphology, strong stereoscopic impressions, round and smooth surface, and no obvious particle–particle adhesion. Figure 3A demonstrates a representative 3D-printed titanium alloy porous scaffold, and Figure 3B illustrates a 3D-printed titanium alloy porous scaffold loaded with gelatin and shell-core microspheres. After being lyophilized by a freeze dryer, the gelatin solution formed the gel at low temperature by wrapping the shell-core microspheres and adhering to the surface of the titanium alloy scaffold and inside the pores. Figures 3C,D represent the composite scaffold cross-linked by genipin. Gelatin appears dark blue after cross-linking with genipin.

### 3.2 Characterization of a 3D printed titanium alloy porous scaffold-loaded VEGF/BMP-2 shell core microsphere sustained-release system

**FIGURE 4:**
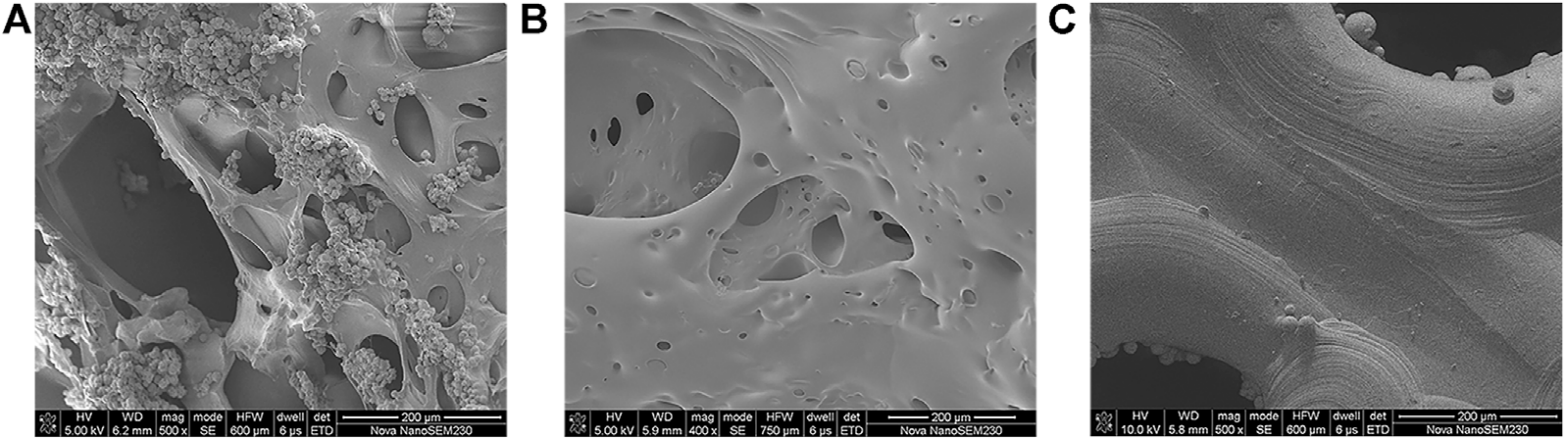
SEM images of **(A)** dual-factor loaded core-shell microspheres-gelatin coating-3D printing titanium alloy porous scaffold; **(B)** gelatin coating-3D printing titanium alloy porous scaffold; **(C)** 3D printed titanium alloy porous scaffolds.

Figure 4 displays the microscopic topography of the scaffolds in each group under SEM. The porous structure was formed by cross-linking of gelatin with many microspheres. Wrapping and adherence were found on the composite scaffold surface of group A, while the gelatin gel coating and the porous structure of gelatin after cross-linking were evident on the composite scaffold surface of group B. Meanwhile, the surface of group C was smoother than that of the other two groups, with the presence of spherical titanium particles.

**FIGURE 5:**
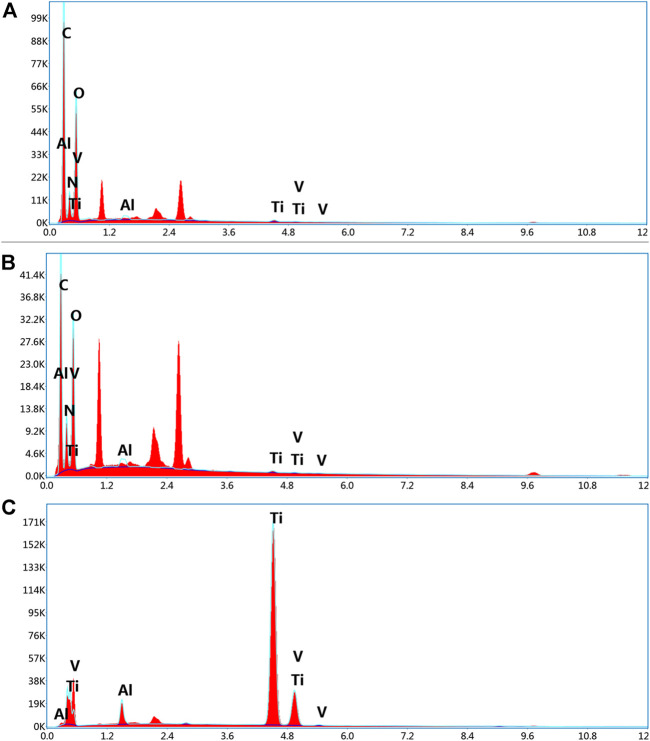
EDS analysis of the surface elemental composition of **(A)** dual-factor loaded core-shell microspheres-gelatin coating-3D printing titanium alloy porous scaffold; **(B)** gelatin coating-3D printing titanium alloy porous scaffold; **(C)** 3D printed titanium alloy porous scaffolds.

Figure 5 demonstrates that the EDS analysis was on the scaffold surface of each group. The chemical formula of PLGA was (C_6_H_8_O_4_)n (C_4_H_4_O_4_)m, and PLLA was (C_6_H_8_O_4_)n, comprised of the elements carbon (C), hydrogen (H), and oxygen (O). Meanwhile, the chemical formula of gelatin was C_10_H_151_N_31_O_39_, and the constituent elements were C, H, O, and nitrogen (N), whereas titanium alloys comprised titanium (Ti), aluminium (Al), and vanadium (V). In addition, Group A consisted of PLGA/PLLA shell-core microspheres-gelatin coating-titanium alloy, with seven elements of C, H, O, N, Ti, Al and V on the surface; Group B was gelatin coated titanium alloy, with the same seven elements of C, H, O, N, Ti, Al, and V on the surface; Group C was titanium alloy, with only three elements (Ti, Al and V) on the surface.

**FIGURE 6:**
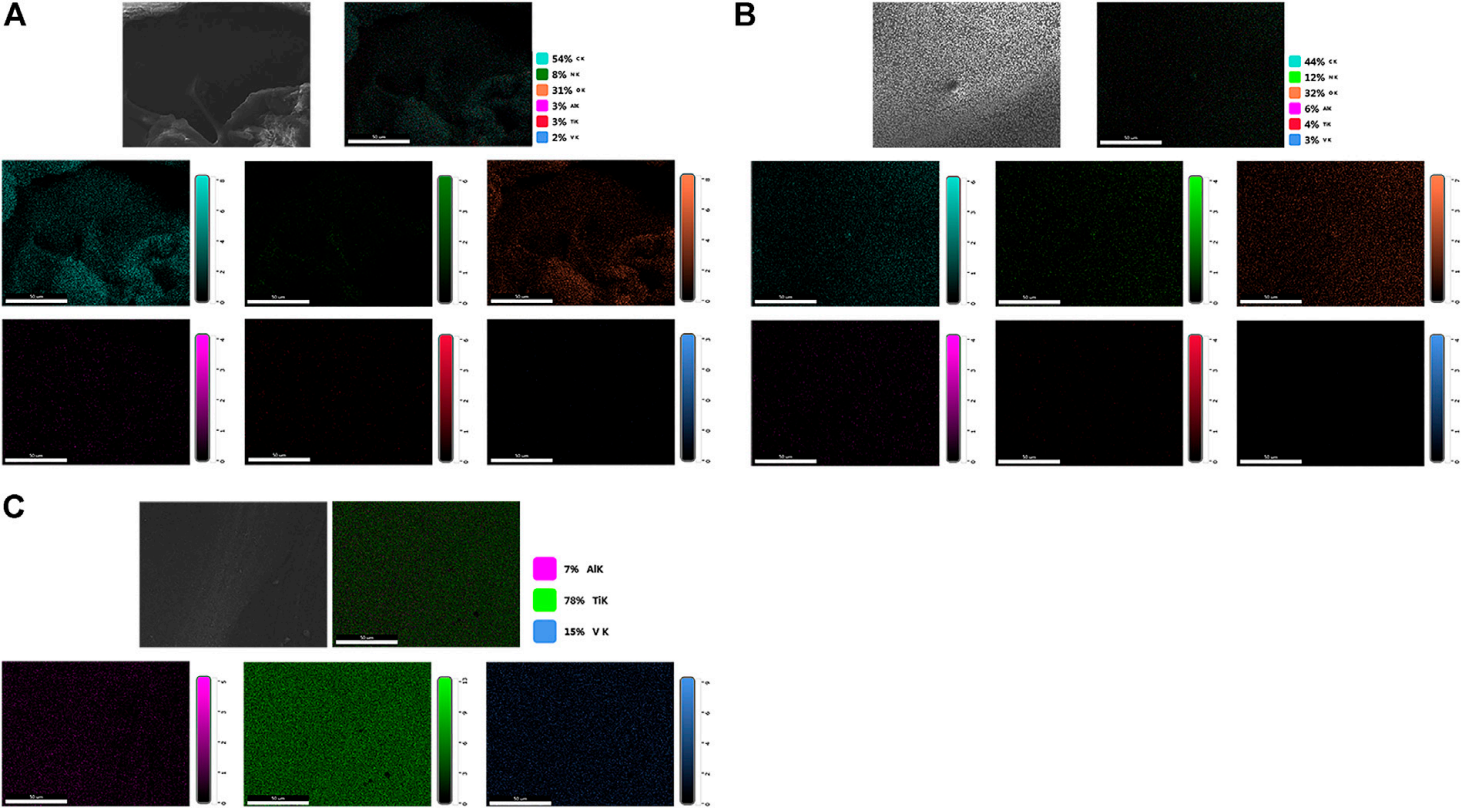
EDS mapping analysis of the surface elemental distribution of **(A)** dual-factor loaded core-shell microspheres-gelatin coating-3D printing titanium alloy porous scaffold; **(B)** gelatin coating-3D printing titanium alloy porous scaffold; **(C)** 3D printed titanium alloy porous scaffolds.

As shown in Figure 6, the scaffolds of each group were detected using EDS mapping. The results demonstrated that the proportions of Al, Ti, and V on the scaffold surface in groups A and B were lower, and the surface distribution was less, which could be attributed to the PLGA/PLLA shell-core microspheres-gelatin coating on the surface of the titanium alloy scaffolds in group A. The N element on the surface of group B was higher than that of group A, which could be attributed to the fact that N element was the component of gelatin, while the proportion of N element in group A was reduced due to the coverage of PLGA/PLLA shell-core microspheres.

**FIGURE 7:**
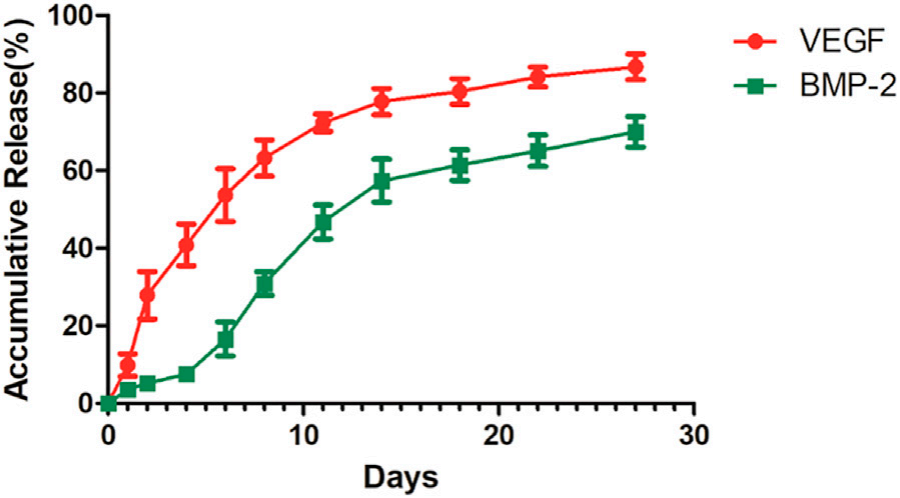
The release curve of 3D-printed titanium alloy porous scaffold loaded with two-factor core-shell microspheres sustained-release system.

Based on Figure 7, the VEGF in the shell exhibited explosive release in the initial stage, while the release rate of both growth factors decreased in the later stage.

### 3.3 Observation of cell morphology on the scaffold surface by scanning electron microscope

**FIGURE 8:**
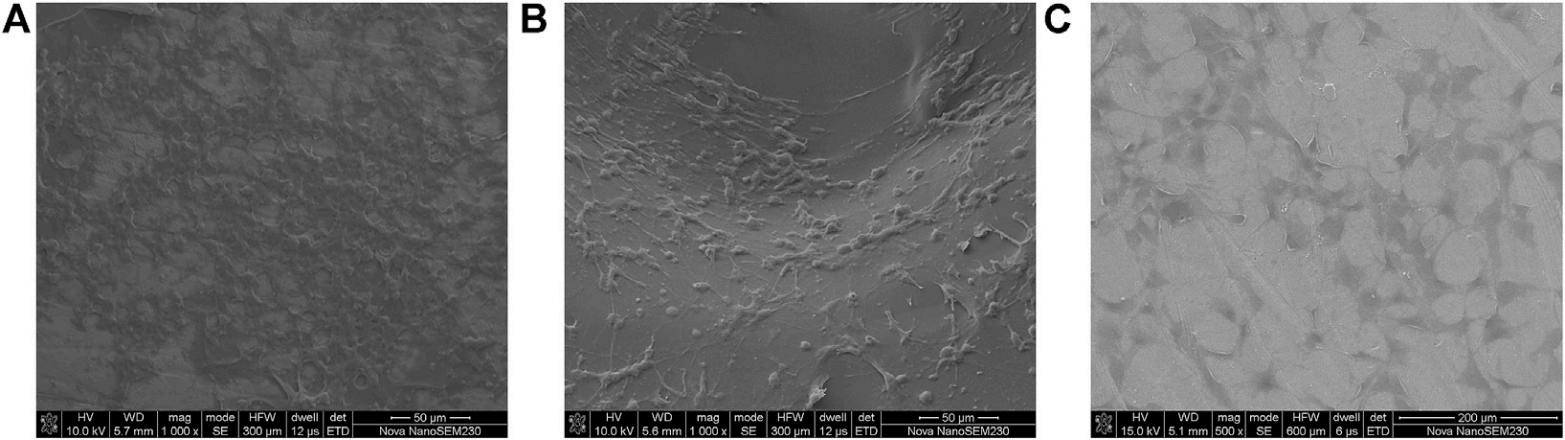
Cell morphology under SEM on **(A)** dual-factor loaded core-shell microspheres-gelatin coating-3D printing titanium alloy porous scaffold; **(B)** gelatin coating-3D printing titanium alloy porous scaffold; **(C)** 3D printed titanium alloy porous scaffolds.

After 48 h, the scaffolds in each group were inoculated with cells, and many cells adhered to the composite scaffold surface in groups A and B. Furthermore, the cells were tightly connected, and the cell pseudopodia were connected with excellent cell spreading (Figure 8). Furthermore, the scaffold cells in group A were highly abundant and appeared stacked, fused, and spread. In contrast, fewer cells adhered to the scaffold surface in group C, which was more dispersed. It was observed that the gelatin coating could promote cell adhesion and growth, while the gelatin coating loaded with growth factor microspheres exhibited improved biocompatibility and stimulated cell adhesion.

### 3.4 Observation of cell morphology on the scaffold surface by confocal laser microscopy

**FIGURE 9:**
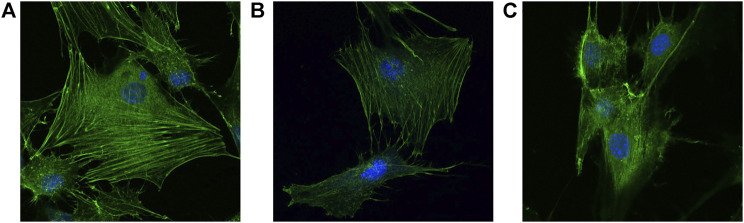
Cell morphology double stainned with DAPI and penicrin under CLSM (×400) on the surface of **(A)** dual-factor loaded core-shell microspheres-gelatin coating-3D printing titanium alloy porous scaffold; **(B)** gelatin coating-3D printing titanium alloy porous scaffold; **(C)** 3D printed titanium alloy porous scaffolds.

Double staining was performed 24 h after the scaffolds of each group were seeded with cells. The cell morphology and cytoskeleton were observed under CLSM. FITC-labeled phalloidin could specifically bind to F-actin in eukaryotic cells, thus displaying the distribution of the actin microfilament skeleton in cells (green fluorescence). In addition, DAPI could bind to DNA in living cells, making the nucleus blue fluorescent. Cells on groups A and B composite scaffolds had more pseudopodia with better cell spread and apparent microfilament arrangement than the pure titanium alloy scaffold of group C (Figure 9). In addition, the fluorescence of cell microfilaments in group A was stronger than in group B, indicating the presence of more microfilaments in group A. Therefore, the gelatin coating loaded with growth factor microspheres exhibited higher biocompatibility and could promote cell adhesion.

### 3.5 Detection of cell proliferation by CCK-8

**FIGURE 10:**
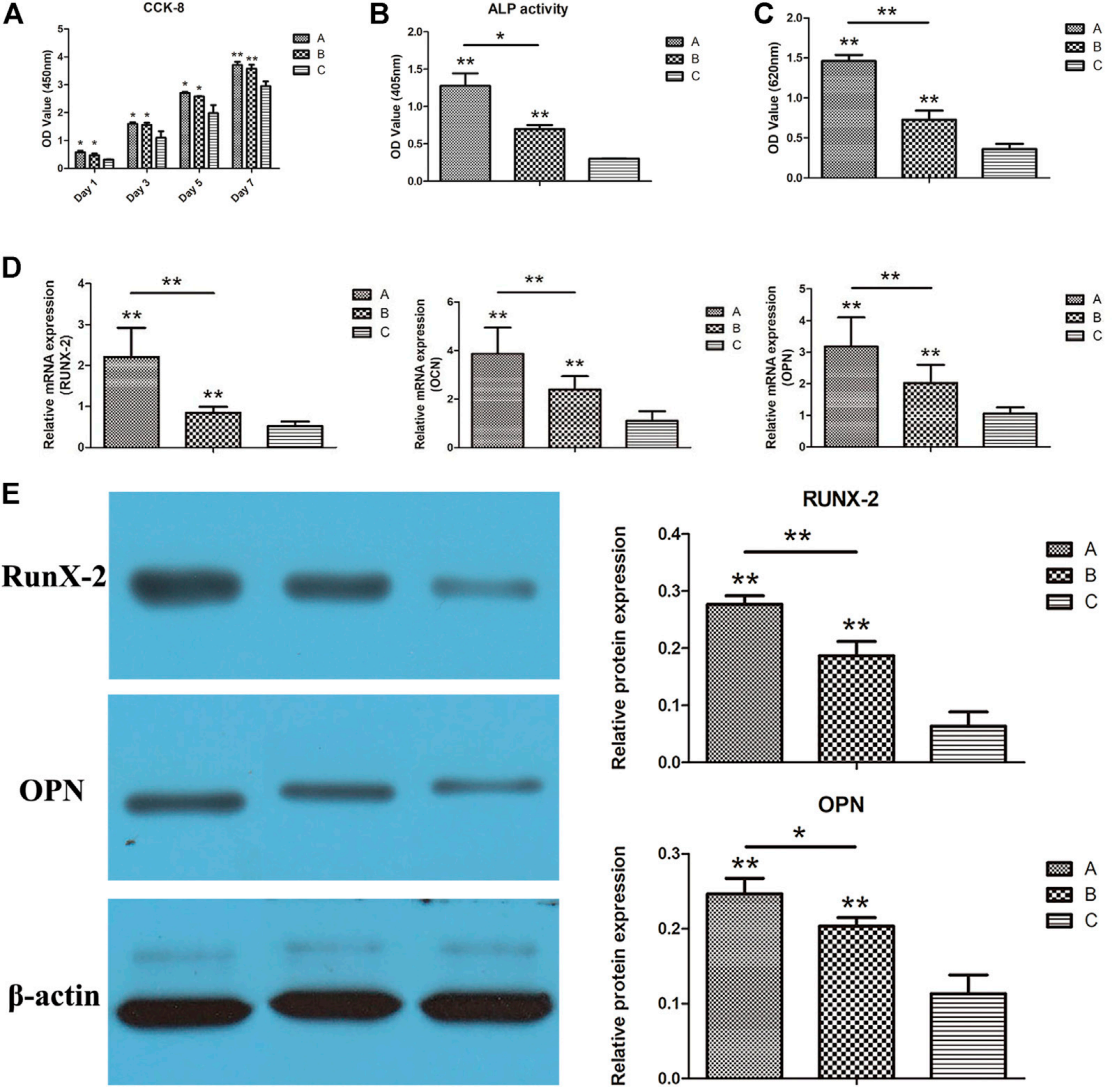
**(A)** CCK-8 results of each group; **(B)** ALP activity results of each group; **(C)** quantitative results of alizarin red staining of each group; **(D)** RUNX-2, OCN, and OPN gene expressions in each group were detected using real-time qPCR; **(E)** the expressions of RUNX-2 and OPN in each group detected using Western blotting. *p < 0.05, **p < 0.01.

The proliferation results of scaffold cells in each group are displayed in Figure 10A. The number of cells on the scaffold in each group revealed a consistently increasing trend with co-culture time. On the 1st, 3rd, and 5th days after co-culture, the number of cells in groups A and B was significantly higher than in groups C (*p* < 0.05). Similarly, the number of cells in groups A and B remained significantly higher than in group C on the 7th day after co-culture, indicating a higher significant level (*p* < 0.01). Meanwhile, there was no significant difference in cell counts between groups A and B (*p* > 0.05) at different time points.

### 3.6 Results of ALP activity test

ALP activities of scaffold cells in each group are shown in Figure 10B. On the 12th day of co-culture, the ALP activities of the cells in groups A and B were significantly higher than in group C (*p* < 0.01). Furthermore, ALP activities of cells in group A were higher than those in group B (*p* < 0.05), indicating that the gelatin coating and the putamen microspheres loaded with bifactors facilitated the promotion of osteogenic differentiation. On top of that, the high ALP activities of cells in group A indicated that gelatin coating and VEGF/BMP-2 loaded shell-core microspheres could be combined to promote bone differentiation.

### 3.7 Quantitative detection results of alizarin red staining

The quantitative results of the alizarin red staining of the scaffold cells in each group are displayed in Figure 10C. On the 21st day after co-culture, the OD values of groups A and B were significantly higher than group C (*p* < 0.01), while the OD values of cells in group A were higher than those in group B (*p* < 0.05). These findings are consistent with ALP activity, suggesting that the gelatin coating and double factor-loaded putamen microspheres can promote the osteogenic differentiation of cells, and the superimposed use can improve the bone-promoting effect.

### 3.8 Detection of osteogenesis-related genes

The real-time qPCR results on the osteogenesis-related gene expressions (RUNX-2, OCN, and OPN) are shown in Figure 10D. Expressions of all three genes in composite scaffold groups A and B were significantly higher than in the control group (*p* < 0.01). Furthermore, the gene expressions in group A were significantly higher than those in group B (*p* < 0.01). Gelatin coating and bifactor-loaded putamen microspheres could promote the expression of osteogenic genes, with pronounced osteogenic induction effects. In addition, the osteogenic induction effects of gelatin coating and bifactor-loaded putamen microspheres could be superimposed, consistent with the results of ALP detection.

### 3.9 Detection results of osteogenesis-related protein

The Western blot results on the osteogenic-related protein expressions (RUNX-2 and OPN) in scaffold cells of each group are displayed in Figure 10E. Both proteins were significantly higher in composite scaffold groups A and B than in the control group (*p* < 0.01). Meanwhile, OPN expression was significantly higher in group A than in group B (*p* < 0.05), and RUNX-2 expression was highly significant in group A than in group B (*p* < 0.01). These findings demonstrated that both gelatin coating and VEGF/BMP-2 shell-core microspheres could promote the expression of osteogenic related proteins, with prominent osteogenic induction effects. In addition, the osteogenic induction effects of gelatin coating and VEGF/BMP-2 shell-core microspheres could be superimposed, consistent with the qPCR results.

### 3.10 *In vivo* experiment of rabbit femoral defect repair

**FIGURE 11:**
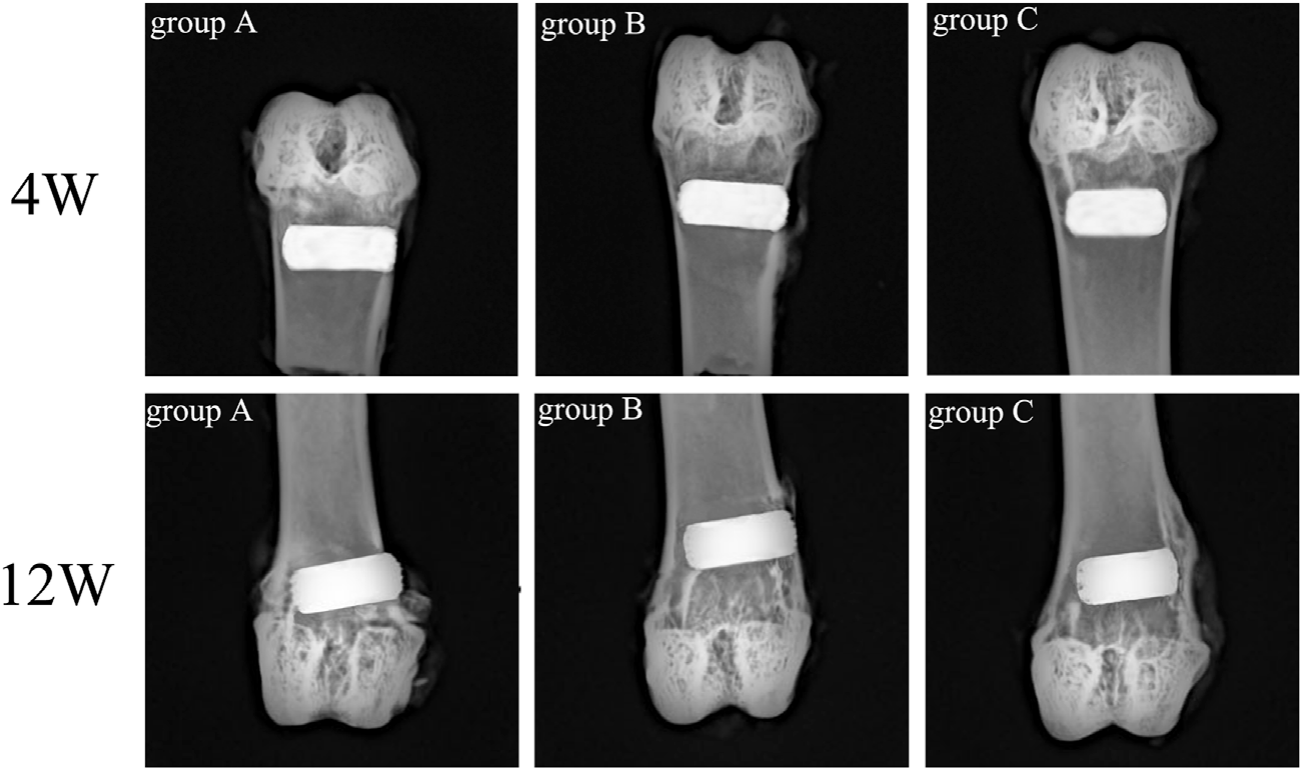
X-ray examination of rabbit femur after scaffold implantation in each group.

Rabbit femur samples were obtained on the 4th and 12th week after scaffold implantation, and an X-ray plane inspection was performed after fixation. Figure 11 illustrated that all implanted scaffolds were in a good position, without loosening, falling, or dislocating. Also, no inflammatory reaction and infection were detected in the samples.

**FIGURE 12:**
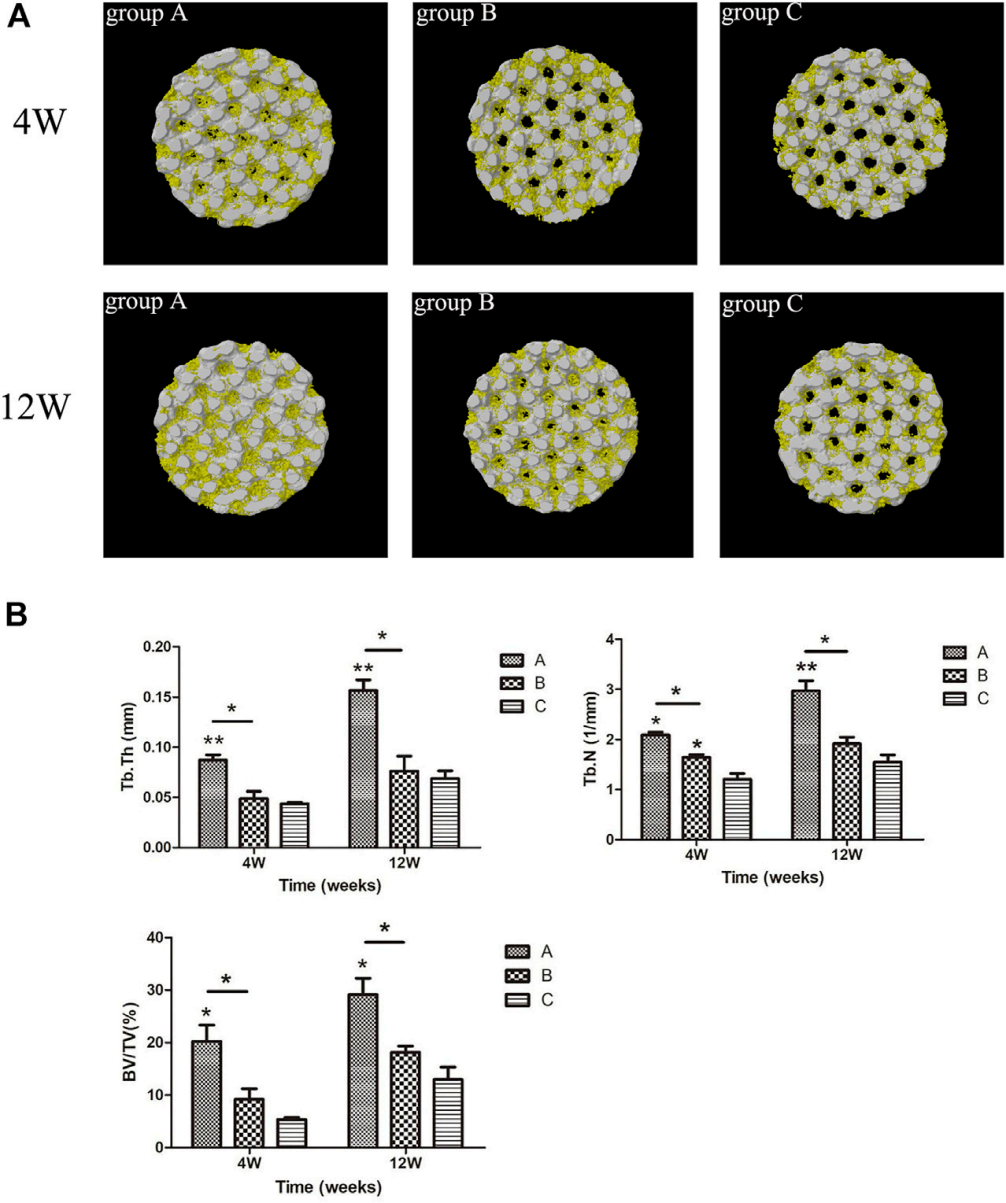
**(A)** Micro-CT 3-D reconstruction results of scaffolds in each group. Yellow = bone tissue, white = titanium porous scaffold; **(B)** micro-CT scanning of new bone tissue parameters in each group. *p < 0.05, **p < 0.01.

The micro-CT scan results were 3D reconstructed with Ctan software to observe the bone ingrowth within the scaffold. Based on Figure 12A, the white part represented the titanium alloy porous scaffold, and the yellow part represented the ingrowth of new bone tissue. The 3D image of the porous scaffold bottom surface exhibited a small amount of new bone tissue growth around the pores in group C and less bone tissue growing into the inner holes at week four. In contrast, new bone tissue was more visible in group B than in group C but with lower ingrowth tissue near the middle. Meanwhile, the amount of new bone tissue in group A was significantly higher than in groups B and C, and a large amount of bone tissue growth was observed in the internal holes. In the 12th week, the amount of new bone in the scaffold of each group increased compared to the 4th week. The amount of new bone in the scaffold of group A was significantly higher than the other two groups, while the amount of new bone in the scaffold of group B was higher than that of group C.

The new bone tissue parameters of the scaffolds in all groups are revealed in Figure 12B. At weeks four and 12, the parameters of new bone tissue in group A were significantly improved compared to the other groups. For instance, Tb.Th in group A was higher than that in group B (*p* < 0.05) and group C (*p* < 0.01). Similarly, BV/TV in group A was higher than in group B (*p* < 0.05) and group C (*p* < 0.05). Meanwhile, Tb.N in group A was higher than in group B (*p* < 0.05, *p* < 0.01, 12 weeks). At week four, Tb.N in group B was higher than in group C (*p* < 0.05), and there was no significant difference in other parameters between group B and group C (*p* > 0.05). Nevertheless, the results for all parameters in group B were higher than in group C.

**FIGURE 13:**
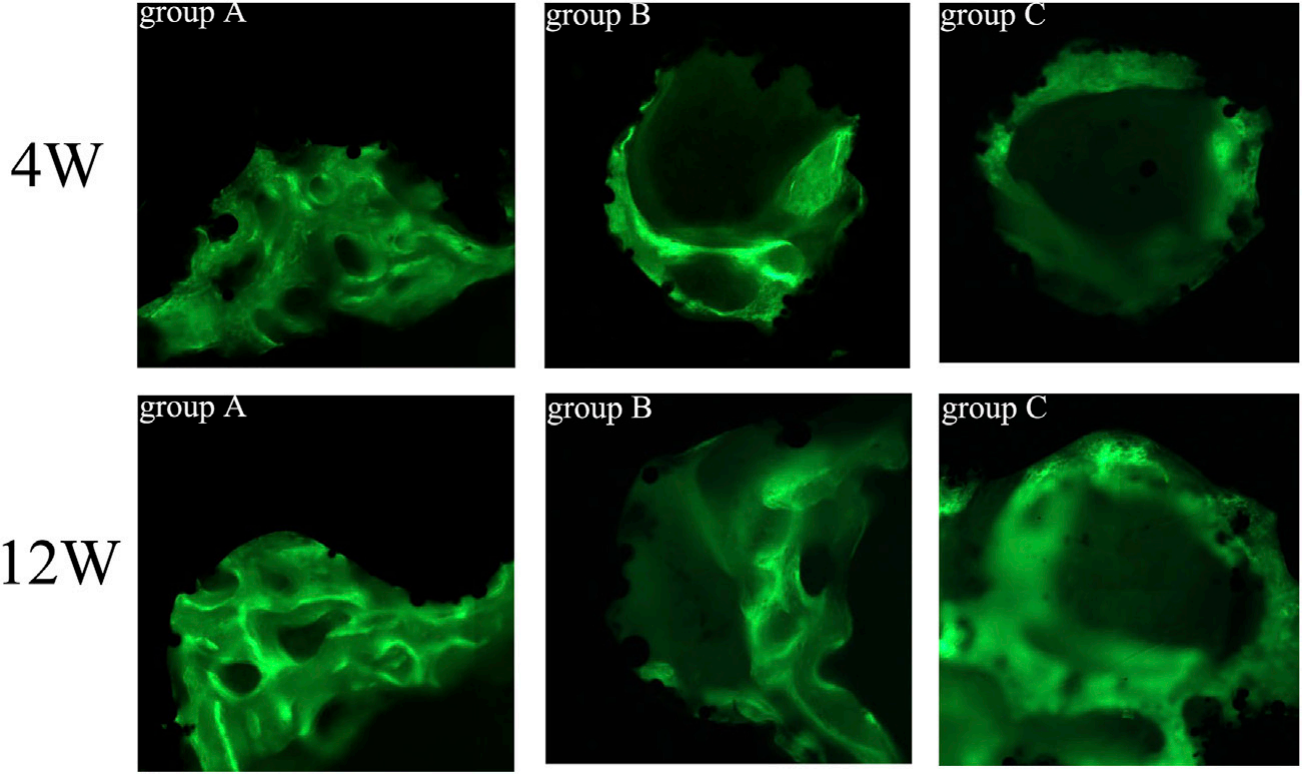
Fluorescence labeling of new bone tissue in porous scaffolds of each group (×200). Green = calcein-labeled new bone tissue, black = titanium alloy.

All hard tissue specimens were sectioned and placed under CLSM to observe the fluorescent labeling of bone tissue (Figure 13). Green represents bone tissue formation marked by calcein, while black represents titanium alloy. In the 4th week, the fluorescent markers in group A indicated mature new bone, and the width of bone mass was significantly higher than in groups B and C. In addition, the mass bone width was higher, and the fluorescence distribution was denser in group B than in group C. At the 12th week, the amount of newly formed bone increased, and the fluorescence distribution was denser in all groups compared to the 4th week. Additionally, the width and thickness of the new bone tissues in group A were significantly greater than in groups B and C, while the width of the new bone tissue in group B was greater than in group C.

**FIGURE 14:**
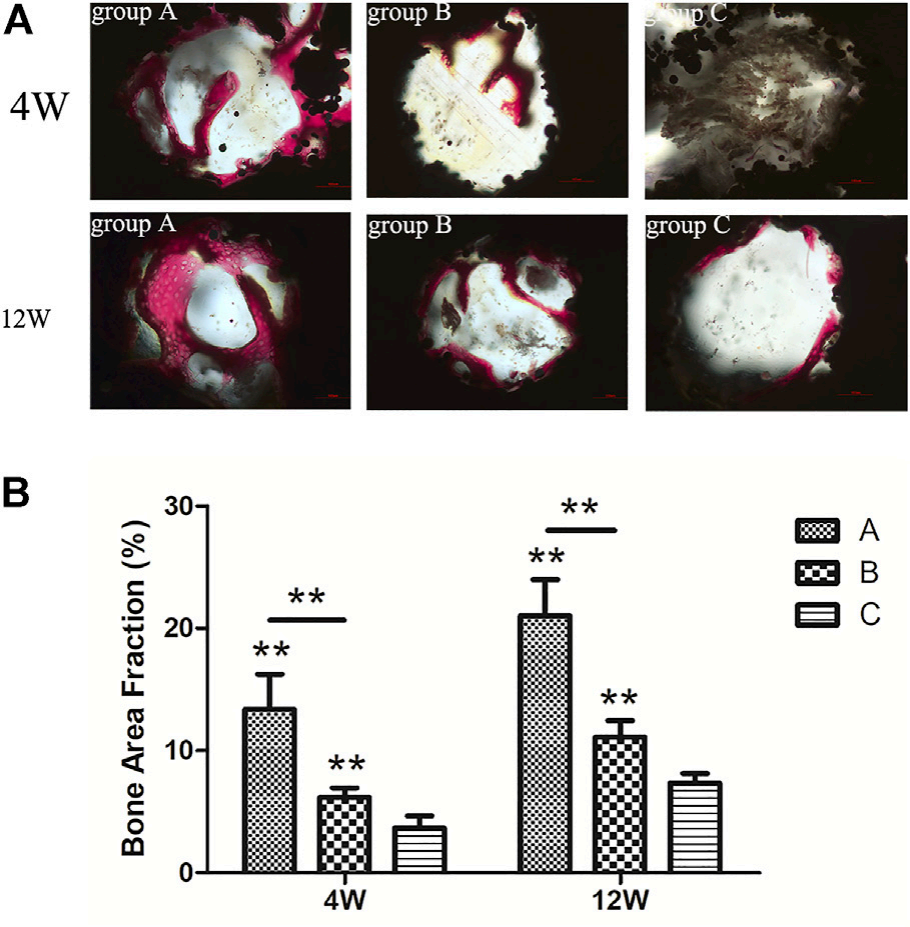
**(A)** VG staining of new bone tissue in each group (×200). Red = mature new bone tissue, brown grey = bone marrow tissue, black = titanium alloy material; **(B)** percentage of the new bone area in titanium porous scaffolds of each group.

All hard tissue sections were stained with VG after CLSM and observed under an optical microscope (Figure 14A). The red color represented mature new bone tissue, brown-grey indicated bone marrow tissue, and black represented the titanium alloy. In the 4th week, more mature new bone tissue grew into the scaffold in group A, structurally firm and compact and adhered closely to the surface of the scaffold. Furthermore, the new bone formation was significantly higher in group A than in groups B and C. On the other hand, the amount of new bone in group B was less than that in group A. However, the new bone shape was mature and dense, with a large amount of bone marrow tissue growth in the scaffold of group C, with less mature bone tissue.

In the 12th week, the bone mass increased in all groups compared to the 4th week. In group C, the bone marrow tissue in the scaffold was substantially reduced, and the mature bone tissue had increased substantially. The bone mass was reduced, and many gaps were observed in the scaffold. In contrast, the new bone mass was higher in group B than in group C, which closely adhered to the material surface with few gaps. Ultimately, new bone mass with compact and regular structures was highly evident in group A compared to groups B and C. The percentage of new bone area in all groups is demonstrated in Figure 14B. At the 4th and 12th week, the new bone area in group A was significantly larger than in groups B (*p* < 0.01) and C (*p* < 0.01), and the new bone area in group B was significantly larger than in group C (*p* < 0.01).

## 4 Discussion

One of the critical challenges in the current orthopedics regenerative medicine is the design of bone scaffolds and implants capable of replicating the host bone biomechanical properties. The porous titanium and associated alloys have become essential in repairing or replacing bone defects since the hardness and porosity of these metals allow for tuning. Furthermore, the unique features of these metals provide an open space for bone tissue to grow, thus, accelerating the osseointegration process (Spriano et al., 2018). Recently, 3D-printed titanium alloy porous scaffolds prepared *via* SLM have gained traction in the field of orthopedics (Wang et al., 2018).

The pore shape in the traditional scaffolds is random, while the titanium alloy porous scaffolds prepared using SLM have a controllable 3D-layered porous structure (Gorgin Karaji et al., 2017; Hedayati et al., 2017). This feature ensures the interconnection of pores necessary for cell growth and nutrient transportation, besides adjusting the mechanical properties, particularly represented by Young’s modulus, to minimize the stress shielding and obtain good mechanical properties (Cheng et al., 2012; Casalino et al., 2015). Nevertheless, the inherent biological inertness of titanium alloy in the scaffold material does not cause bone regeneration, thus limiting the usage of this material for specific orthopedic treatments, such as the repair of segmental bone defects (Li et al., 2017). On the other hand, the tight bond between the porous titanium alloy and bone tissue ensures good long-term osseointegration. Various studies have performed surface modification on porous titanium alloys to improve osseointegration (Li et al., 2019). The surface changes of porous titanium alloy, such as surface roughness, biomaterial coating or growth factors, can improve the biological activity of the material surface, induce and enhance bone regeneration, and ultimately reduce bone integration time (Chen et al., 2020; Thukkaram et al., 2020).

Gelatin is one of the preferred polymers for manufacturing various biological materials. The bioactivity of gelatin that supports cell adhesion and interaction with signal molecules is crucial for cell transmission and tissue regeneration. In addition, the physicochemical properties of gelatin, especially the adjustable cross-linking density, degradation kinetics, and gelling properties, provide high usability for the design of drug delivery vehicles. The synergistic use of gelatin and other biomaterials enables greater flexibility in material degradation and drug release while maintaining and enhancing the properties of the matrix material. Gelatin is easily absorbed by host tissues after implantation due to the high biocompatibility and hydrophilicity, in addition to the absence of antigenic expression under physiological conditions (Bae et al., 2018). Gelatin coating on the surface of the porous titanium alloy provides a suitable growth environment for osteoblasts, which can improve initial cell adhesion, osteoblast proliferation, and differentiation (Vanderleyden et al., 2014; Costa et al., 2017). Animal studies that utilize rabbit and goat femurs demonstrated significantly increased bone growth and bone implant contact around titanium implants coated with gelatin (Bernhardt et al., 2005; Morra et al., 2005).

Gelatin is known to exhibit rapid dissolution in aqueous environments. However, the cross-linking achieved by physical or chemical methods can improve the physical and biochemical stability of gelatin in the aqueous phase, prolong the degradation time, and further reduce antigenicity by linking antigen epitopes to avoid swallowing or being recognized by the immune system (Kim et al., 2021). The physical cross-linking methods of gelatin include microwave irradiation, dehydrogenation heat treatment, and UV treatment; the main advantage of physical methods is the absence of potential hazards, whereas the main disadvantage is the difficulty in obtaining the desired degree of cross-linking. Common chemical crosslinkers for gelatin contain aldehydes (formaldehyde, glutaraldehyde, glyceraldehyde), polyhydroxy compounds, and carbodiimides. However, most of these cross-linking agents are synthetic and cytotoxic, resulting in unreacted cross-linking agents remaining in the cross-linked scaffold. Consequently, removing residual toxicity is challenging, thus, leading to the formation of toxic substances during biodegradation *in vivo* (Tonda-Turo et al., 2011). Therefore, the present study adopted genipin as the cross-linking agent for gelatin.

Genipin is the product of geniposide hydrolyzed by β-glucosidase and is an excellent natural biological cross-linking agent with lower toxicity than glutaraldehyde and other commonly used chemical cross-linking agents. Furthermore, by cyclic cross-linking of natural biomaterials, genipin can generate a deep blue pigment called “gardenia blue” (Somers et al., 2008). Thus, the surface of the gelatin-coated composite scaffold cross-linked by genipin in the current study appeared dark blue. The current SEM results demonstrated that the cross-linked gelatin has a porous structure, with the pores exhibiting high connectivity. Meanwhile, the combination of the porous structure of the cross-linked gelatin hydrogel coating with the porous titanium alloy scaffold could significantly increase the permeability of the composite scaffold, promote the exchange of nutrients and metabolites between cells and the surrounding physiological environment, and facilitate angiogenesis and bone growth.

The PLGA/PLLA putamen-loaded VEGF/BMP-2 VEGF/BMP-2 sustained-release microsphere with excellent biocompatibility was fabricated in this research, useful in simulating bone repair by releasing growth factors and promoting osteogenic differentiation *in vivo* (Wang et al., 2015). The 3D-printed titanium alloy porous scaffold-loaded VEGF/BMP-2 shell-core microsphere sustained-release system was successfully prepared by adhering and wrapping the VEGF/BMP-2 shell-core microspheres with a gelatin hydrogel coating to promote the close adhesion with the 3D-printed titanium alloy porous scaffold. Based on the *in vitro* release experiment, the findings indicated that the general trend of the release curve was consistent with that of the VEGF (shell)/BMP-2 (core) microspheres alone. On day 10, the cumulative release of VEGF exceeded 60% of the total amount and the cumulative release of BMP-2 was approximately 40%. An explosive VEGF release in the shell was evident at the initial stage but remained slower than that of microspheres alone, and the cumulative release of VEGF was less than 30% on the 2nd day. The above situation could be attributed to the fact that some microspheres are completely coated with gelatin, leading to a relatively prolonged degradation time. The release rates of VEGF and BMP-2 were slower in later stages, consistent with the release curve of microspheres alone.

The cytoskeleton was observed *via* CSLM, and the cells in the composite scaffold had more pseudopodia, spreading, and prominent microfilament arrangement than in the pure titanium alloy scaffold. Furthermore, the fluorescence of cell microfilaments in group A was stronger than in group B, indicating the higher presence of cell microfilaments in group A. These findings indicated that the microspheres loaded with growth factors and gelatin-coating have good biocompatibility, and the combined application can promote cell adhesion. Furthermore, the results of CCK-8 cell proliferation did not exhibit statistical differences between combined application and gelatin coating alone.

The ALP activity is an early marker of osteoblast differentiation. Enhanced activity of ALP may enhance bone formation and bone matrix mineralization. In the osteogenic differentiation experiment, the ALP quantitative detection reflected the differentiation level of osteoblasts. The higher activity suggested higher differentiation of osteogenic precursor cells to mature osteoblasts. Furthermore, RUNX-2 is now recognized as a transcription factor necessary for osteoblast differentiation and an essential regulator during osteogenic differentiation, while OPN and OCN are common osteogenic markers for differentiation. Various indicators of osteogenic differentiation demonstrated that both composite scaffolds could significantly upregulate the gene expression of RUNX-2, OCN, and OPN and significantly promoted ALP, RUNX-2, and OPN proteins. Moreover, gelatin hydrogel-coated scaffolds loaded with growth factor microspheres better facilitated osteogenic differentiation of osteoblast precursor cells than the simple gelatin hydrogel-coated scaffolds.

In summary, the evidence found in the *in vitro* studies was verified by the *in vivo* experiments. The micro-CT, fluorescence labeling of hard tissue sections, and VG staining results implied that the gelatin-coated titanium alloy porous scaffolds loaded with growth factor microspheres had more new bone mass, closer contact with the surface of scaffolds, and stronger ability to promote bone differentiation and integration than the gelatin-coated titanium alloy porous scaffolds and simple titanium alloy porous scaffolds.

## 5 Conclusion

The sustained-release microspheres loaded with VEGF/BMP-2 shell-core prepared in this study were loaded on a 3D-printed titanium alloy porous scaffold through gelatin hydrogel coating to achieve the sequential release of VEGF and BMP-2. Furthermore, the *in vitro* and *in vivo* findings demonstrated that the system could effectively promote osteogenic differentiation and osseointegration. This work provides experimental results as well as a strategy for the repair of bone defects.

## Data Availability

The original contributions presented in the study are included in the article/supplementary material, further inquiries can be directed to the corresponding author.
